# Relationship between Novel Anthropometric Indices and the Prevalence of Abdominal Aortic Calcification: A Large Cross-Sectional Study

**DOI:** 10.31083/j.rcm2412349

**Published:** 2023-12-13

**Authors:** Yanwei Yin, Hanzhi Wu, Fangmeng Lei, Wenlin Lu, Yanqing Shen, Wenjing Hu, Xiaoxiao Liu, Xinhe Ye, Chengjian Yang

**Affiliations:** ^1^Department of Cardiology, Wuxi No.2 People’s Hospital, 214000 Wuxi, Jiangsu, China; ^2^Department of Cardiology, Wuxi No.2 People’s Hospital, Wuxi Clinical College of Nanjing Medical University, 214000 Wuxi, Jiangsu, China

**Keywords:** abdominal aortic calcification, body roundness index, a body shape index, U.S. population, cross-sectional study

## Abstract

**Background:**

The relationship between novel anthropometric indices, specifically a body shape index (ABSI) and body roundness index (BRI), with abdominal aortic calcification (AAC) or severe AAC (SAAC) is unclear. The aim of our study was therefore to investigate possible relationships between novel anthropometric indices and prevalence of AAC and SAAC.

**Methods:**

We obtained U.S. general population data from the National Health and Nutrition Examination Survey between 2013 and 2014. The study used restricted cubic spline (RCS) analysis, multivariable logistic regression modeling, subgroup analysis, and receiver operating characteristic (ROC) curve assessment. We investigated relationships between ABSI or BRI and AAC and SAAC risk. Associations between ABSI or BRI and the degree of AAC were also evaluated using a generalized additive model.

**Results:**

The study cohort was comprised of 1062 individuals. The RCS plots revealed a U-shaped curve associating ABSI with AAC risk. A similar trend emerged for SAAC, where the risk initially increased before subsequently decreasing with rising ABSI levels. Additionally, BRI exhibited a positive correlation with both AAC and SAAC risk. As ABSI and BRI values increased, the degree of AAC also increased. In ROC analysis, ABSI displayed a significantly larger area under the curve compared to BRI.

**Conclusions:**

ABSI is associated with AAC prevalence following a U-shaped curve. Additionally, BRI is positively correlated with AAC risk. ABSI demonstrates a superior discriminative ability for AAC compared to BRI. Therefore, maintaining an appropriate ABSI and BRI may reduce the prevalence of AAC.

## 1. Introduction 

Vascular calcification refers to the pathological buildup of apatite mineral deposits in the vascular system [1]. This process primarily affects the abdominal aorta, the coronary artery, the femoral artery, the thoracic aorta, and the carotid artery [2]. Notably, the calcification of the abdominal aorta, known as abdominal aortic calcification (AAC) serves as an early indicator of atherosclerosis in this artery [3]. The prevalence and progression of AAC are closely linked to several conventional cardiovascular risk factors, including age, gender, and smoking [4, 5]. Epidemiological studies have highlighted the potential of using spine radiographs to gauge the severity of AAC as an effective approach for evaluating cardiovascular morbidity and mortality [6, 7, 8, 9, 10].

Anthropometric indices serve as the primary diagnostic and screening tools for identifying obesity in individuals. These indices include body weight, waist circumference (WC), and body mass index (BMI). Due to their effectiveness, noninvasiveness, and ease of application, these measures are widely adopted in clinical practice [11]. While BMI is commonly used to evaluate general obesity, it lacks accuracy in assessing body fat distribution and the true obesity status of individuals. Recent studies emphasize the heightened impact of abdominal obesity compared to general obesity. To comprehensively evaluate diverse obesity patterns, a combination of BMI and WC assessment is essential [12]. In recent years, two new anthropometric indices have been developed, namely and body roundness index (BRI) and a body shape index (ABSI) [13, 14]. ABSI is calculated by adjusting WC for height and weight, and thus provides a more precise depiction of central abdominal adiposity [15]. Additionally, BRI can be used to predict both body fat and the percentage of visceral adipose tissue [16]. Previous studies have highlighted the association of ABSI with hypertension, diabetes mellitus (DM), and cardiovascular disease, with stronger links to mortality than either BMI or WC [17]. Moreover, Xu *et al*. [18] concluded that BRI had superior predictive ability and stronger associations with cumulative cardiometabolic risk factors compared to BMI and WC. Nonetheless, data on the correlation between novel anthropometric indices (ABSI and BRI) and prevalence of AAC remains scarce. Therefore, the aim of this study was to explore the connections between ABSI, BRI, and AAC prevalence in adults using data from the U.S. National Health and Nutrition Examination Survey (NHANES). This should provide valuable clinical insight for preventing and intervening in AAC.

## 2. Materials and Methods

### 2.1 Study Population

**Fig. 1.**
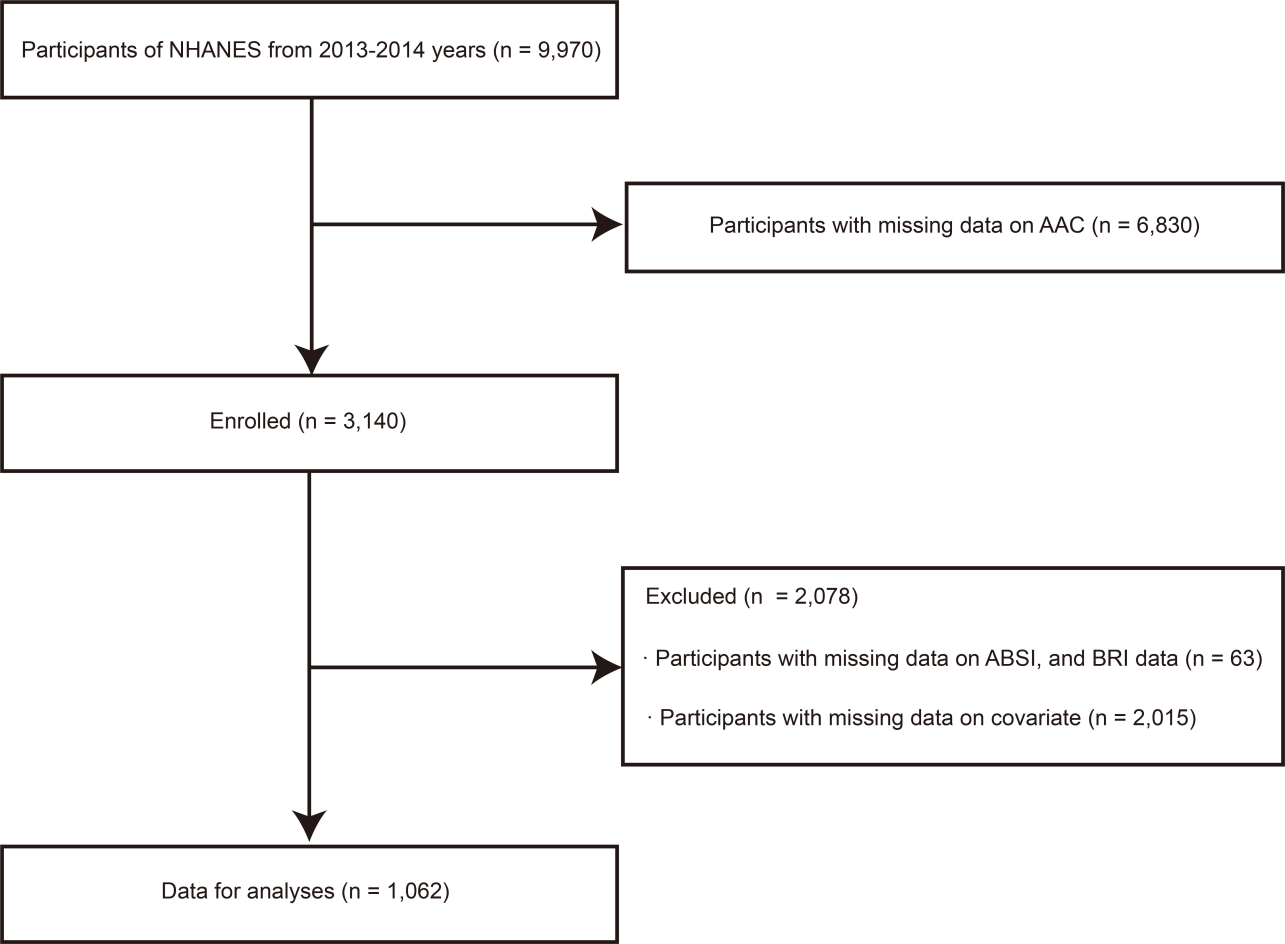
**NHANES participants in the current study**. Abbreviations: AAC, abdominal aortic calcification; ABSI, a body shape index; BRI, body roundness index; NHANES, National Health and Nutrition Examination Surveys.

The NHANES used a stratified, multistage and random sampling design. It is a nationally representative and cross-sectional survey conducted on the United States (U.S.) civilian, non-institutionalized resident population. The present study was based on data collected between 2013 and 2014 by the NHANES survey (Fig. 1). A total of 9970 participants were included, with AAC data available for 3140. Participants with missing data for the novel anthropometric indices (a body shape index [ABSI] and body roundness index [BRI]) were excluded (n = 63). Also excluded were participants with missing covariate data (n = 2015), leaving a final total of 1062 participants in the study. An informed consent form was provided to all participants in the NHANES study. All protocols were approved by the National Center for Health Statistics Research Ethics Review Board [19]. The NHANES website provides more information about the survey design, methods, population, and data (https://www.cdc.gov/nchs/nhanes/about_nhanes.htm).

### 2.2 Anthropometric Measurements

Basic anthropometric measurements were made by experienced examiners. These used standardized techniques and equipment and included body height (BH), weight and WC. Participants were measured barefoot and in light clothing, with BH and weight being reported to the nearest 0.1 cm and 0.1 kg, respectively. With the participant standing, a flexible anthropometric tape was used to measure the WC midway between the lowest rib and the iliac crest. The following formulae were used to calculate ABSI and BRI [20]:

$$\mathrm{ABSI}=\mathrm{WC}\,\left(m\right)/{\mathrm{BMI}}^{2/3}\,\left(\mathrm{kg}/m^{2}\right)\times {\mathrm{BH}}^{1/2}\,\left(m\right)$$

$$\mathrm{BRI}=364.2-365.5\times \sqrt{1-{\left(\frac{\frac{W\,C\,\left(m\right)}{2\,\pi }}{0.5\times B\,H\,\left(m\right)}\right)}^{2}}$$

### 2.3 AAC Measurement

To obtain and quantify AAC, trained and certified radiology professional technologists performed the dual-energy X-ray absorptiometry (Densitometer Discovery A, Hologic, Marlborough, MA, USA). And, Kauppila score systems were conducted on the lumbar spine (vertebrae L1–L4) [21, 22]. The higher the AAC score, the more severe the calcification of the abdominal aorta. A range of 0 to 24 was used for the Kauppila score in this study. An AAC score equal to 0 had no calcification, an AAC score greater than 0 and less than or equal to 6 had mild-moderate calcification, and an AAC score greater than 6 had severe calcification [23, 24, 25].

### 2.4 Covariates

The following covariates were included in this study: sex, age, education level, family poverty income ratio (PIR), race/ethnicity, marital status, drinking status, smoking status, complications from DM, and hypertension, complications from congestive heart failure (CHF), angina pectoris, stroke, coronary heart disease (CHD), and heart attack, BMI, systolic blood pressure (SBP), WC, dietary phosphorus intake, diastolic blood pressure (DBP), dietary calcium intake, mean energy intake, fasting blood glucose (FBG), total bilirubin, glycohemoglobin (HbA1c), hemoglobin (Hb), fast insulin, alkaline phosphatase, serum phosphorus and calcium, high-density lipoprotein-cholesterol (HDL-C), uric acid (UA), total cholesterol (TC), serum creatinine (Scr), triglyceride (TG), estimated glomerular filtration rate (eGFR), and blood urea nitrogen (BUN).

### 2.5 Statistical Analysis

All analyses were performed using R software (version 4.2.0, R Foundation for Statistical Computing, Vienna, Austria) and SPSS software (version 22.0, IBM SPSS statistics, Chicago, IL, USA). A *p*-value of <0.05 was regarded as statistically significant. ABSI, BRI and BMI were divided into quartiles, with the lowest quartile group (Q1 group) serving as the reference group. Data for continuous variables are presented as the mean and standard deviation (SD), and data for categorical variables as numbers (%). Differences between groups were calculated using weighted *T*-tests for continuous variables and weighted chi-square tests for categorical variables. The NHANES sample weights were taken into account when calculating all estimates. Multivariate logistic regression analysis was performed to investigate links between ABSI, and BRI and prevalence of AAC and severe AAC (SAAC). Model 1 was adjusted for sex and age. Model 2 was adjusted for the model 1 variables plus drink status, smoke status, education level, family PIR, race or ethnicity, marital status, and complications from DM, and hypertension. Finally, model 3 was adjusted for all variables in Table 1.

**Table 1.** **Demographic characteristics of the study participants**.

Variables		Overall (n = 1062)	Non-AAC (n = 723)	AAC (n = 339)	*p*-value
Age, years		58.16 ± 0.50	55.71 ± 0.45	64.02 ± 1.08	<0.001
Sex, %					0.804
	Male	518 (48.8%)	352 (33.1%)	166 (15.6%)	
	Female	544 (51.2%)	371 (34.9%)	173 (16.3%)	
Race, %					0.538
	Mexican American	137 (12.9%)	99 (9.3%)	38 (3.6%)	
	Other Hispanic	89 (8.4%)	63 (5.9%)	26 (2.4%)	
	Non-Hispanic Black	191 (18.0%)	139 (13.1%)	52 (4.9%)	
	Non-Hispanic White	519 (48.9%)	330 (31.1%)	189 (17.8%)	
	Other race	126 (11.9%)	92 (8.7%)	34 (3.2%)	
Family PIR		3.22 ± 0.15	3.33 ± 0.13	2.97 ± 0.19	0.017
Marital status, %					0.004
	Having a partner	714 (67.2%)	510 (48.0%)	204 (19.2%)	
	No partner	265 (25.0%)	153 (14.4%)	112 (10.5%)	
	Unmarried	83 (7.8%)	60 (5.6%)	23 (2.2%)	
Education level, %					0.072
	Less than high school	216 (20.3%)	145 (13.7%)	71 (6.7%)	
	High school	219 (20.6%)	142 (13.4%)	77 (7.3%)	
	More than high school	627 (59.0%)	436 (41.1%)	191 (18.0%)	
Hypertension, %					<0.001
	No	489 (46.0%)	380 (35.8%)	109 (10.3%)	
	Yes	573 (54.0%)	343 (32.3%)	230 (21.7%)	
Diabetes mellitus, %					0.005
	No	800 (75.3%)	571 (53.8%)	229 (21.6%)	
	Yes	262 (24.7%)	152 (14.3%)	110 (10.4%)	
Smoker, %					0.013
	No	561 (52.8%)	413 (38.9%)	148 (13.9%)	
	Former	317 (29.8%)	188 (17.7%)	129 (12.1%)	
	Now	184 (17.3%)	122 (11.5%)	62 (5.8%)	
Alcohol user, %					0.462
	No	139 (13.1%)	97 (9.1%)	42 (4.0%)	
	Former	231 (21.8%)	145 (13.7%)	86 (8.1%)	
	Mild	417 (39.3%)	289 (27.2%)	128 (12.1%)	
	Moderate	135 (12.7%)	100 (9.4%)	35 (3.3%)	
	Heavy	140 (13.2%)	92 (8.7%)	48 (4.5%)	
CHD, %					0.010
	No	1007 (94.8%)	699 (65.8%)	308 (29.0%)	
	Yes	55 (5.2%)	24 (2.3%)	31 (2.9%)	
CHF, %					0.028
	No	1024 (96.4%)	707 (66.6%)	317 (29.8%)	
	Yes	38 (3.6%)	16 (1.5%)	22 (2.1%)	
Angina pectoris, %					0.155
	No	1028 (96.8%)	704 (66.3%)	324 (30.5%)	
	Yes	34 (3.2%)	19 (1.8%)	15 (1.4%)	
Heart attack, %					<0.001
	No	1006 (94.7%)	702 (66.1%)	304 (28.6%)	
	Yes	56 (5.3%)	21 (2.0%)	35 (3.3%)	
Stroke, %					0.006
	No	1013 (95.4%)	702 (66.1%)	311 (29.3%)	
	Yes	49 (4.6%)	21 (2.0%)	28 (2.6%)	
Hyperlipidemia, %					0.066
	No	241 (22.7%)	187 (17.6%)	54 (5.1%)	
	Yes	821 (77.3%)	536 (50.5%)	285 (26.8%)	
BMI, kg/$m^{2}$		28.52 ± 0.32	28.96 ± 0.29	27.47 ± 0.47	0.003
Waist circumference, cm		99.68 ± 0.74	100.06 ± 0.62	98.78 ± 1.37	0.309
SBP, mmHg		124.37 ± 0.93	122.61 ± 0.71	128.56 ± 2.04	0.001
DBP, mmHg		69.48 ± 0.57	70.67 ± 0.58	66.66 ± 0.89	<0.001
Mean energy intake (kcal/day)		2017.08 ± 38.90	2050.69 ± 38.83	1936.99 ± 62.66	0.044
Dietary calcium intake, mg		928.82 ± 15.83	953.79 ± 19.44	869.30 ± 26.65	0.018
Dietary phosphorus intake, mg		1355.43 ± 17.15	1386.50 ± 18.13	1281.40 ± 31.49	0.006
Hemoglobin, g/dL		14.30 ± 0.06	14.31 ± 0.08	14.28 ± 0.07	0.778
Fast glucose, mg/dL		107.51 ± 1.23	106.32 ± 1.27	110.34 ± 2.49	0.152
Fast insulin, pmol/L		72.76 ± 4.72	74.14 ± 5.83	69.48 ± 6.40	0.575
HbA1c, %		5.76 ± 0.04	5.70 ± 0.05	5.89 ± 0.08	0.074
Alkaline phosphatase, U/L		0.68 ± 0.01	0.68 ± 0.01	0.69 ± 0.03	0.713
Total bilirubin, g/dL		65.68 ± 0.96	65.07 ± 1.29	67.12 ± 2.21	0.482
Phosphorus, mg/dL		3.73 ± 0.02	3.73 ± 0.03	3.73 ± 0.03	0.967
TC, mg/dL		195.93 ± 1.29	196.23 ± 1.75	195.21 ± 2.07	0.735
Calcium, mg/dL		9.41 ± 0.01	9.39 ± 0.02	9.46 ± 0.03	0.085
HDL-C, mg/dL		55.62 ± 0.72	56.01 ± 0.75	54.68 ± 1.19	0.262
TG, mg/dL		124.46 ± 3.36	124.07 ± 3.68	125.38 ± 4.66	0.782
Uric acid, mg/dL		5.47 ± 0.07	5.42 ± 0.06	5.58 ± 0.18	0.341
eGFR, mL/min/1.73 $m^{2}$		84.75 ± 0.76	88.15 ± 0.83	76.66 ± 1.75	<0.001
Blood urea nitrogen, mg/dL		13.96 ± 0.22	13.44 ± 0.20	15.18 ± 0.50	0.001
Serum creatinine, mg/dL		0.92 ± 0.01	0.88 ± 0.01	1.01 ± 0.04	0.008
ABSI		0.083 ± 0.000	0.082 ± 0.00	0.084 ± 0.00	<0.001
BRI		5.42 ± 0.10	5.36 ± 0.16	5.44 ± 0.09	0.007

Abbreviations: ABSI, a body shape index; BRI, body roundness index; BMI, body mass index; CHD, coronary heart disease; CHF, congestive heart failure; DBP, diastolic blood pressure; eGFR, estimated glomerular filtration rate; HDL-C, high-density lipoprotein-cholesterol; HbA1c, glycosylated hemoglobin; SBP, systolic blood pressure; TC, total cholesterol; TG, triglycerides; PIR, poverty income ratio; AAC, abdominal aortic calcification.

## 3. Results

### 3.1 Baseline Characteristics

The baseline characteristics for the research participants are presented in Table 1. In this study cohort the incidence of AAC and SAAC was 31.9% and 11.7%, respectively. The participants in this study represent an estimated 44,977,275 individuals in the US population. Participant characteristics were subclassified according to ABSI quartiles (Q1: 0.068–0.080; Q2: 0.081–0.083; Q3: 0.084–0.086; Q4: 0.087–0.108), BRI quartiles (Q1: 1.436–4.096; Q2: 4.097–5.267; Q3: 5.268–6.642; Q4: 6.643–13.267) and BMI quartiles (Q1: 15.400–24.625; Q2: 24.626–27.800; Q3: 27.801–31.300; Q4: 31.301–50.400). Significant differences were found between the Non-AAC and AAC groups in terms of age, family PIR, marital status, complications from hypertension and DM, smoking, the complication of CHF, heart attack, CHD, stroke, BMI, SBP, DBP, dietary phosphorus and calcium intake, mean energy intake, Scr, eGFR, BUN and ABSI.

### 3.2 Associations between ABSI, BRI or BMI with AAC and SAAC

**Fig. 2.**
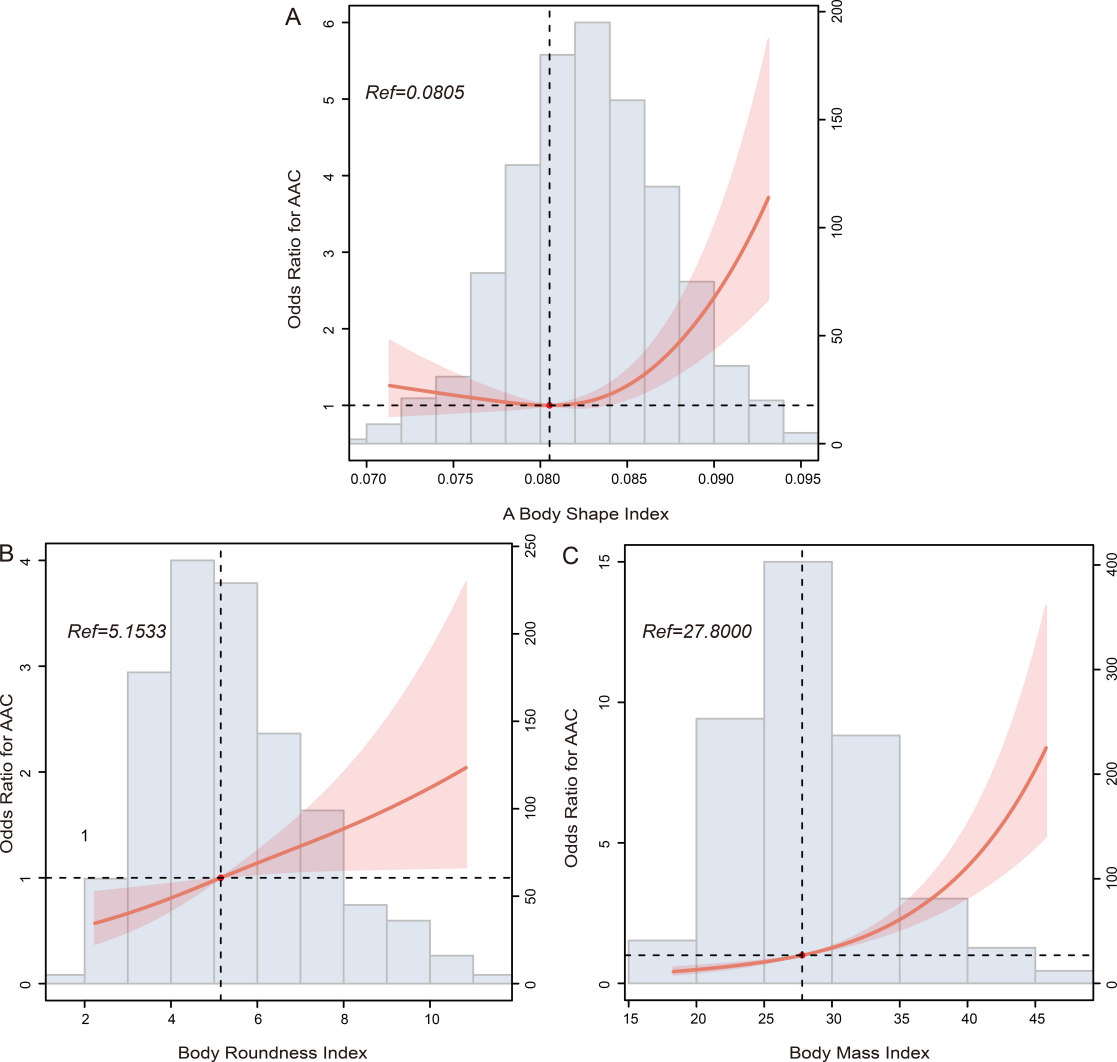
**RCS curves showing the relationships between AAC and (A) a body shape index, (B) body roundness index, and (C) body mass index **. Abbreviations: AAC, abdominal aortic calcification; RCS, restricted cubic spline.

The relationship between ABSI and BRI with the prevalence of AAC and SAAC was explored using multivariate logistic regression analysis, as presented in Tables 2,3. The restricted cubic spline (RCS) plot revealed a U-shaped association between ABSI and AAC prevalence (*p* for nonlinearity = 0.013, Fig. 2A). As ABSI increased, the prevalence of AAC decreased significantly. This decline was most pronounced when ABSI reached 0.0805, followed by an upward trend in prevalence.

**Table 2.** **Associations of ABSI, and BRI with the prevalence of AAC**.

	Model 1		Model 2		Model 3	
	OR (95% CI)	*p* for trend	OR (95% CI)	*p* for trend	OR (95% CI)	*p* for trend
ABSI		<0.001		0.009		0.364
Q1 (0.068–0.080)	1.00		1.00		1.00	
Q2 (0.087–0.108)	1.079 (0.704, 1.653)		0.989 (0.636, 1.539)		0.968 (0.575, 1.630)	
Q3 (0.084–0.086)	1.549 (1.022, 2.348) *		1.270 (0.821, 1.964)		1.092 (0.584, 2.044)	
Q4 (0.081–0.083)	2.096 (1.365, 3.218) ***		1.698 (1.082, 2.666)		1.453 (0.625, 3.376)	
BRI		0.392		0.020		0.398
Q1 (1.436–4.096)	1.00		1.00		1.00	
Q2 (4.097–5.267)	1.098 (0.741, 1.627)		1.019 (0.677, 1.535)		1.196 (0.717, 1.994)	
Q3 (5.268–6.642)	0.920 (0.619, 1.367)		0.740 (0.484, 1.131)		1.148 (0.603, 2.187)	
Q4 (6.643–13.267)	0.886 (0.594, 1.321)		0.636 (0.407, 0.992) *		1.761 (0.690, 4.492)	

Abbreviations: AAC, abdominal aortic calcification; ABSI, a body shape index; BRI, body roundness index; CI, confidence interval; OR, odds ratio. *p < 0.05, ***p < 0.001; Model 1: sex, and age; Model 2: model 1 variables plus alcohol user, marital status, race/ethnicity, smoker, family poverty-income ratio, education level, the complication of diabetes mellitus, and hypertension; Model 3 was adjusted for all variables in Table 1.

**Table 3.** **Associations of ABSI, and BRI with the prevalence of SAAC**.

	Model 1		Model 2		Model 3	
	OR (95% CI)	*p* for trend	OR (95% CI)	*p* for trend	OR (95% CI)	*p* for trend
ABSI		0.086		0.594		0.554
Q1 (0.068–0.080)	1.00		1.00		1.00	
Q2 (0.087–0.108)	1.495 (0.695, 3.217)		1.377 (0.620, 3.056)		1.083 (0.700, 4.649)	
Q3 (0.084–0.086)	2.365 (1.158, 4.834) *		1.785 (0.839, 3.799)		2.805 (0.723, 6.007)	
Q4 (0.081–0.083)	1.857 (0.899, 3.834)		1.325 (0.617, 2.847)		1.669 (0.417, 6.675)	
BRI		0.470		0.030		0.146
Q1 (1.436–4.096)	1.00		1.00		1.00	
Q2 (4.097–5.267)	1.407 (0.773, 2.560)		1.210 (0.640, 2.286)		2.029 (0.872, 4.725)	
Q3 (5.268–6.642)	1.090 (0.595, 1.996)		0.781 (0.403, 1.515)		2.024 (0.703, 5.827)	
Q4 (6.643–13.267)	0.900 (0.484, 1.674)		0.547 (0.272, 1.102)		3.914 (0.876, 17.484)	

Abbreviations: ABSI, a body shape index; BRI, body roundness index; CI, confidence interval; OR, odds ratio; SAAC, severe abdominal aortic calcification. *p < 0.05; Model 1: sex, and age; Model 2: model 1 variables plus alcohol user, marital status, race/ethnicity, smoker, family poverty-income ratio, education level, the complication of diabetes mellitus, and hypertension; Model 3 was adjusted for all variables in Table 1.

**Fig. 3.**
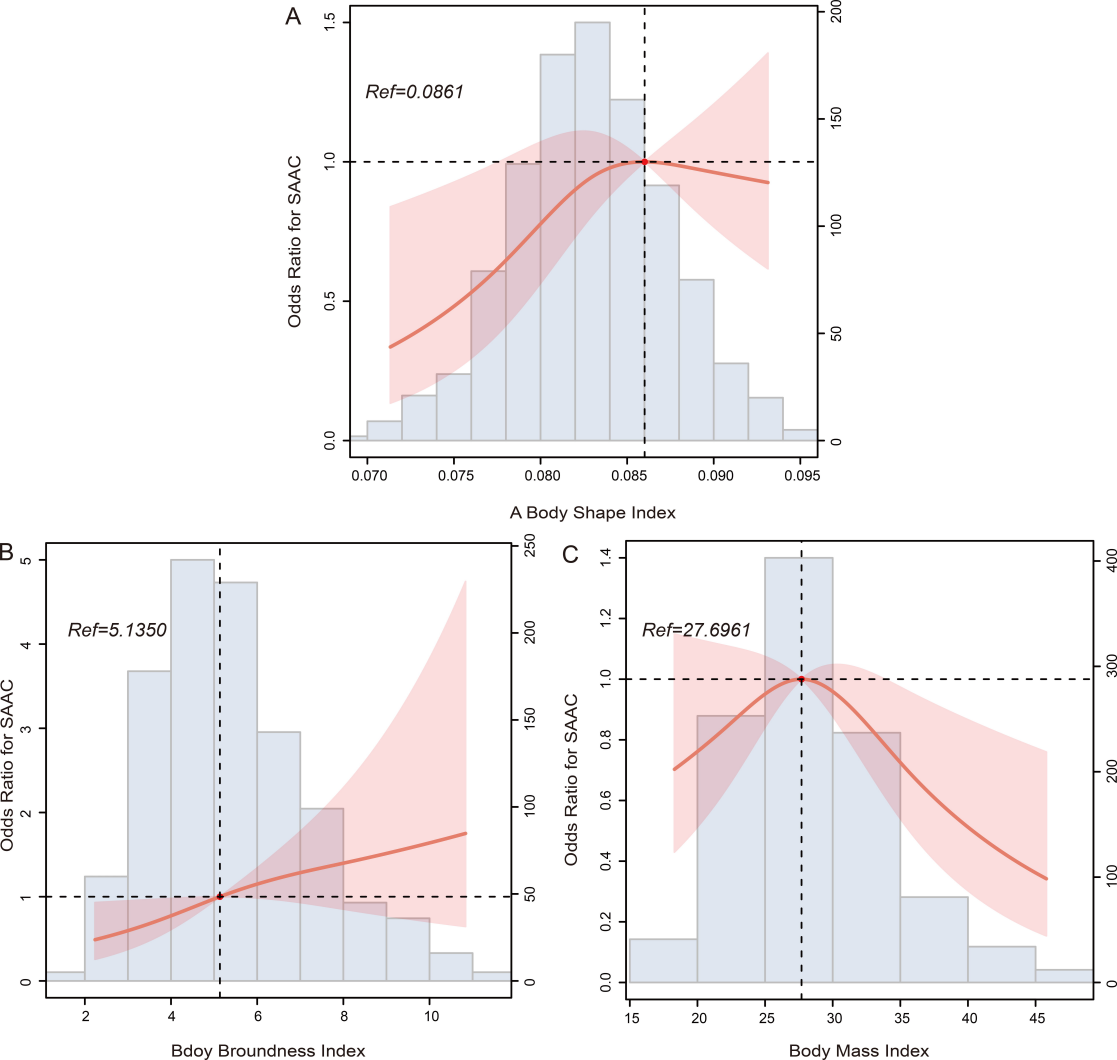
**RCS curves showing the relationships between SAAC and (A) a body shape index, (B) body roundness index, and (C) body mass index**. Abbreviations: RCS, restricted cubic spline; SAAC, severe abdominal aortic calcification.

Similarly, an intriguing pattern emerged for ABSI and BMI concerning SAAC risk, with a trend of initial increase followed by decrease (*p* for nonlinearity = 0.325, Fig. 3A, and *p* for nonlinearity = 0.257, Fig. 3C, respectively). However, both BRI and BMI exhibited a positive correlated with AAC risk (*p* for nonlinearity = 0.659, Fig. 2B, and *p* for nonlinearity = 0.560, Fig. 2C, respectively). Furthermore, BRI displayed a positive correlation with SAAC risk (*p* for nonlinearity = 0.550, Fig. 3B).

### 3.3 Association between ABSI, BRI and BMI with the Degree of AAC

**Fig. 4.**
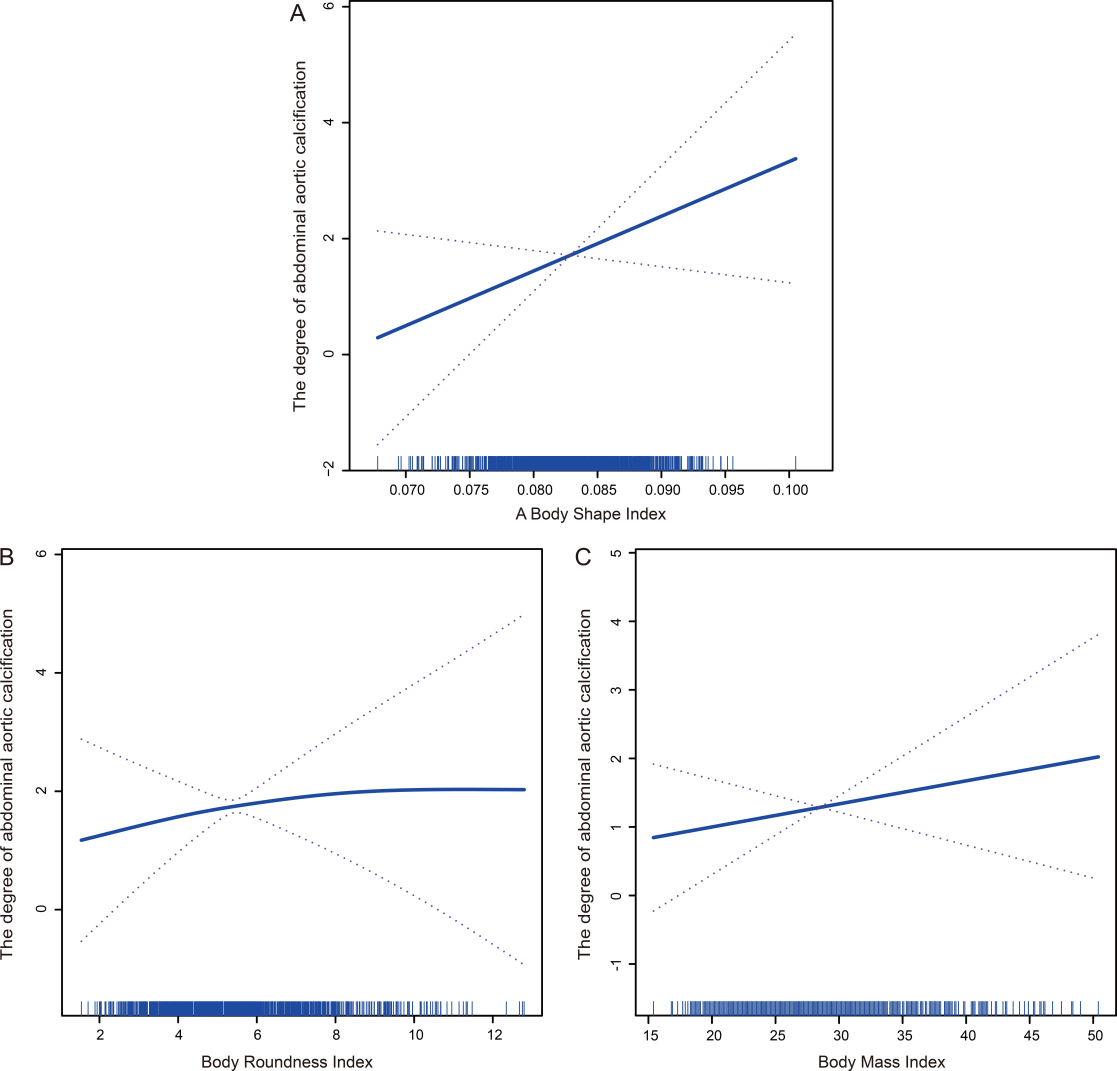
**Associations between the degree of calcification and a body shape index (A), body roundness index (B), and body mass index (C)**.

Based on the findings from the generalized additive models, a consistent linear positive correlation emerged between ABSI, BRI and BMI. As ABSI, BRI and BMI increased, there was a gradual and corresponding increase in the degree of calcification (Fig. 4A–C, respectively).

### 3.4 Discrimination Ability of ABSI, BRI and BMI

**Fig. 5.**
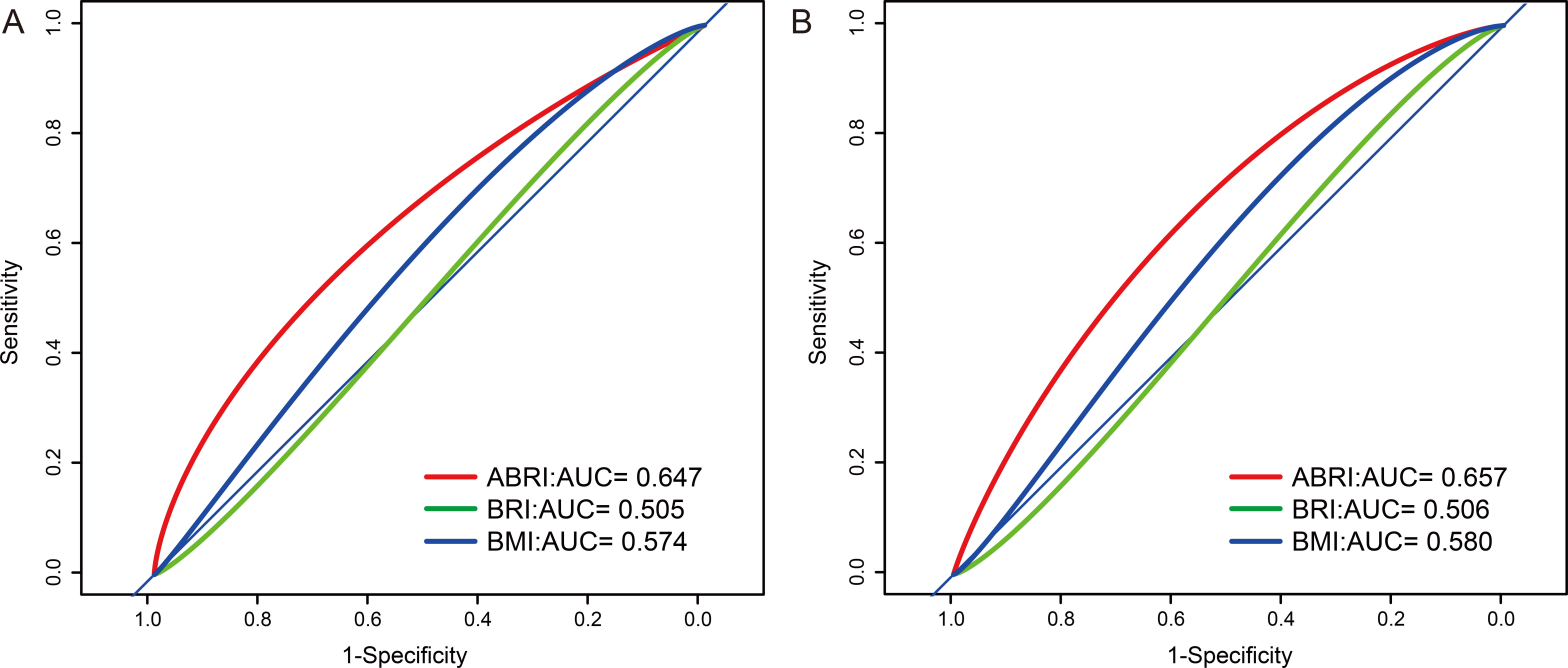
**ABSI, BRI, and BMI used to identify individuals with AAC (A) or SAAC (B)**. Abbreviations: AAC, abdominal aortic calcification; ABSI, a body shape index; AUC, area under the curve; BMI, body mass index; BRI, body roundness index; SAAC, severe abdominal aortic calcification.

To evaluate the capacity of the anthropometric measures (ABSI, BRI, and BMI) to differentiate individuals with AAC from those with SAAC, a receiver operating characteristic (ROC) curve was employed. The ROC curve analysis demonstrated that ABSI outperformed both BRI and BMI in effectively discriminating between AAC (Fig. 5A) and SAAC (Fig. 5B). The optimal threshold values for detection of AAC using ASBI, BRI and BMI were determined to be 0.0836, 3.7696 and 32.10, respectively. Similarly, for identifying SAAC, the optimal cut-off values were 0.0834 for ABSI, 4.1287 for BRI, and 18.90 for BMI.

### 3.5 Subgroup Analyses

Subgroup analysis, stratified by age, sex, hypertension and DM was conducted to investigate the association between ABSI, BRI, and the prevalence of AAC and SAAC (**Supplementary Tables 1,2**). The U-shaped relationship between ABSI and AAC prevalence identified in individuals <60 years old, males and females, individuals with or without hypertension, and individuals without DM (**Supplementary Fig. 1**). Furthermore, the stratified analysis revealed a consistent positive linear correlation between BRI and AAC across all age groups, genders, individuals with or without hypertension, and individuals without DM (**Supplementary Fig. 2**). The scope of subgroup analysis extended to exploring the correlations between ABSI, BRI, and the risk of SAAC (**Supplementary Tables 3,4**; **Supplementary Figs. 3,4**).

## 4. Discussion

This is the first study to examine the novel anthropometric indices (ABSI and BRI) in relation to the prevalence of AAC and SAAC among people from the United States. The key findings of this study include the following: (1) the association between ABSI and AAC prevalence exhibited a U-shaped pattern; (2) individuals with higher BRI values were more likely to experience AAC compared to those with lower BRI values; (3) as ABSI, BRI and BMI increase, the degree of AAC also increases; (4) the discriminative ability of ABSI to predict the risk of both AAC and SAAC surpassed that of BRI and BMI. Based on these findings, we propose that ABSI and BRI may play critical roles in the risk management for AAC and SAAC.

ABSI and BRI are novel anthropometric measurements used to gauge abdominal obesity and visceral adiposity [26]. In previous studies, ABSI demonstrated a positive correlation with central obesity and metabolic-related diseases [13, 27]. Notably, Geraci *et al*. [28] reported that ABSI’s potential as a superior indicator compared to traditional markers (BMI and WC) for predicting carotid atherosclerosis in hypertensive patients. However, ABSI’s predictive value was also found to be similar to conventional risk factors (BMI and WC) for predicting disease risk or death.

Furthermore, two studies conducted on Chinese populations of all ages demonstrated the correlation of ABSI with pre-hypertension or hypertension was no stronger than BMI or WC [29, 30]. Ji *et al*. [17] reported that elevated ABSI levels correlated with a higher risk of hypertension (13%), DM (35%), cardiovascular disease (21%) and overall mortality (55%). While ABSI outperformed weight, height, and BMI in predicting all-cause mortality, its predictive capability for chronic diseases was comparatively weaker [17]. Moreover, among Chinese community adults, Zhang *et al*. [31] identified a positive correlation between ABSI and urinary albumin-creatinine ratio. However, Wei Li *et al*. [32] found the linear positive correlation between ABSI and risk of AAC demonstrated a superior discrimination ability compared to BMI, WC, height, or weight.

A study with a large European cohort observed a J-shaped relationship between BMI, WC and overall mortality, whereas higher ABSI was correlated with higher all-cause mortality [33]. Additionally, the study also highlighted that BMI was a more effective predictor of mortality from cardiovascular disease compared to ABSI [33]. These results are not in agreement with the present study. The risk of disease associated with ABSI has not been previously described as a U-shaped curve. Thus, further studies are needed to confirm that the association between AAC risk associated with ABSI follows the pattern observed in our data. Our study is also the first to show an association between BRI and AAC prevalence.

In a previous study, Tian *et al*. [34] demonstrated that using BRI as a solitary anthropometric measure was more effective in identifying a cluster of cardiometabolic abnormalities in Chinese women. This effectiveness surpassed the discriminative capability of both BMI and the waist-to-height ratio [34]. Zhang *et al*. [35] found that the risk of hypertension increases with the increase of ABSI and BRI in Chinese individuals. Additoanlly, BRI was found to be superior to ABSI in identifying new-onset hypertension. Work by Wu *et al*. [36] further supported an independent positive association between BRI and hypertension risk, aligning with our study’s findings. Finally, Zhou *et al*. [37] demonstrated the relationship between BRI, cardiovascular-related mortality, and overall mortality also followed a U-shaped curve.

While other studies have explored the correlation between BRI and different diseases, to our knowledge BRI has not been compared to other anthropometric measures for its association with AAC. Our study also demonstrated a positive correlation between BMI and AAC prevalence. Bacha reported the process of vascular calcification in obese youths begins in childhood and is primarily driven by obesity [38], aligning with our findings. Yet, Uhlinova *et al*. [39] found that obesity was not an independent predictor of vascular calcification in patients with chronic kidney disease. Finally, our study revealed that ABSI was a stronger independent predictor of AAC, and SAAC, compared to BRI and BMI.

The findings of this study provide further evidence supporting the idea that a more accurate evaluation of AAC risk may be achieved by assessing ABSI and BRI. Nevertheless, our study has several limitations. Firstly, this study’s cross-sectional design restricts the identification of causal relationships between ABSI, BRI AAC, and SAAC. Secondly, while the multivariate logistic regression models adjusted for significant confounding factors, some potential confounding factors including inflammation indicators and medication use were not considered. Thirdly, ABSI and BRI values were highly concentrated around their means, showing only small variance. This circumstance complicates the identification of crucial values with clinical relevance. Finally, this study solely relies on NHANES data from the 2013–2014 U.S. general population, which could limit the applicability of the results to other ethnic populations and nations.

## 5. Conclusions

This study focused on assessing the relationship between ABSI and BRI, and the prevalence of AAC and SAAC. In ABSI, the prevalence of AAC displayed a U-shaped curve, featuring an inflection point at 0.0805, marking the lowest occurrence of AAC. Furthermore, both ABSI and BRI exhibited positively correlations with SAAC risk. A noteworthy finding was that ABSI showcased a stronger discriminative ability in predicting the risk of both AAC and SAAC when compared to BRI and BMI. However, whether ABSI and BRI are suitable for clinical practice requires further studies to determine if our results can be generalized to other ethnic population groups.

## Data Availability

The survey data are publicly available on the Internet for data users and researchers throughout the world https://www.cdc.gov/nchs/nhanes/.

## Supplementary Material

Supplementary material associated with this article can be found, in the online version, at https://doi.org/10.31083/j.rcm2412349.
