# Supplementary material for: Relationship between Novel Anthropometric Indices and the Prevalence of Abdominal Aortic Calcification: A Large Cross-Sectional Study
Source: Rev Cardiovasc Med. 2023 Dec 13;24(12):349. doi: 10.31083/j.rcm2412349 (PMC11272853; doi:10.31083/j.rcm2412349)
Supplement: Supplementary file 1 [file 2153-8174-24-12-349-s1.zip › 2153-8174-24-12-349-s1.docx]

Supplementary Materials


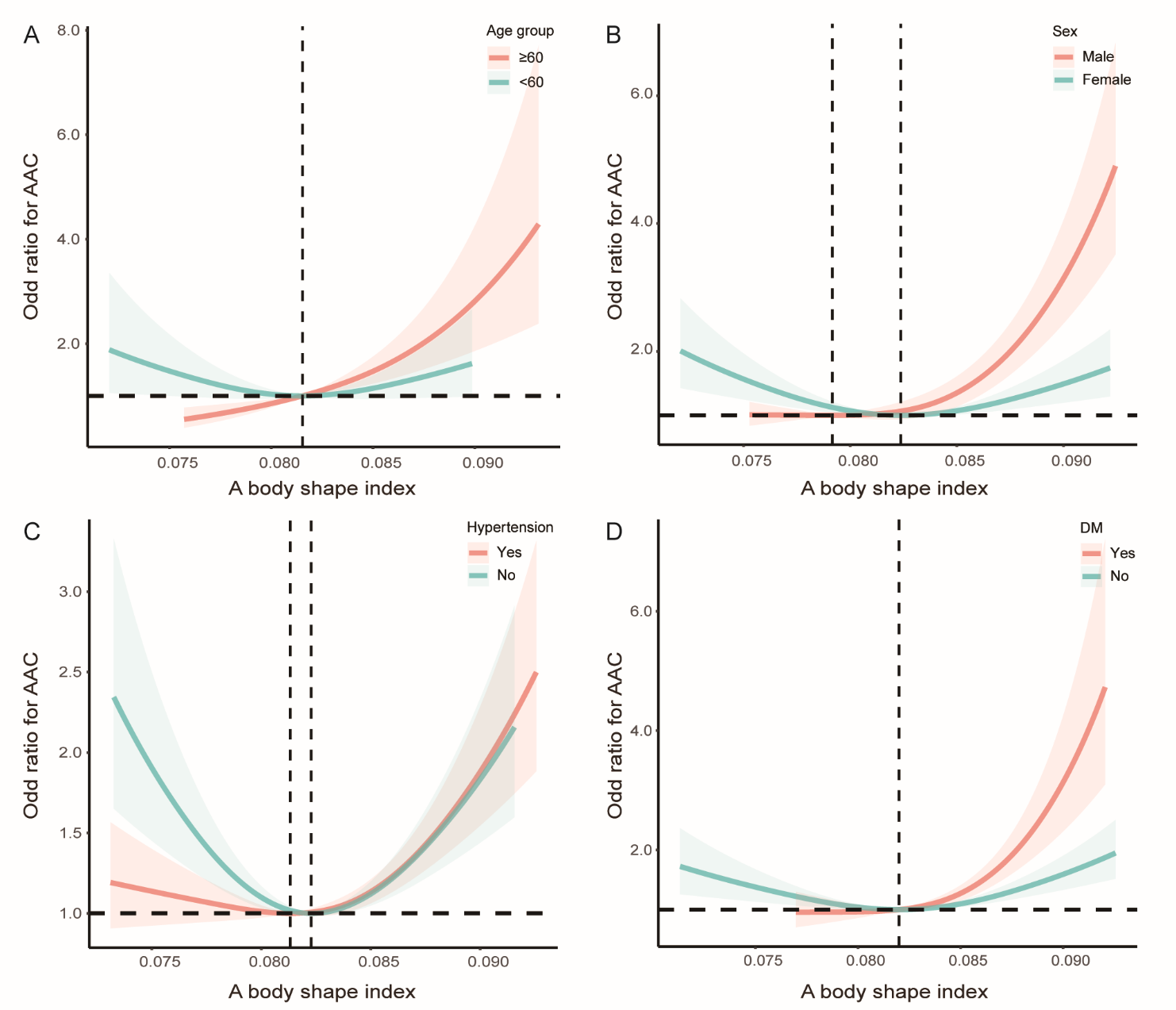


**Supplementary Fig. 1:** The RCS curve of the association between ABSI and AAC stratified by (A) Age, (B) Sex; (C) Hypertension, and (D) DM.

Abbreviation: RCS, restricted cubic spline; ABSI, a body shape index; AAC, abdominal aortic calcification; DM, diabetes mellitus.


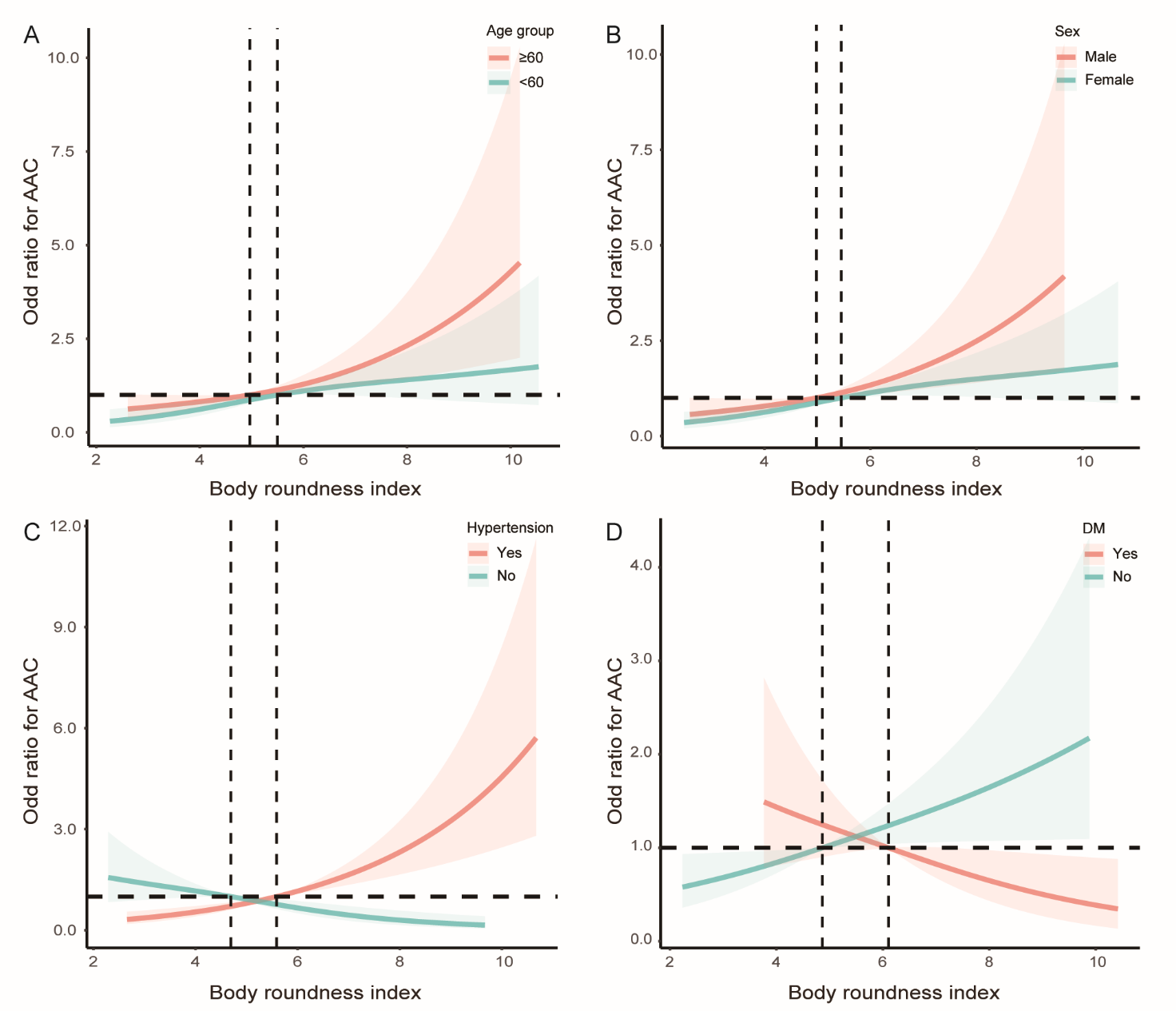


**Supplementary Fig. 2:** The RCS curve of the association between BRI and AAC stratified by (A) Age, (B) Sex; (C) Hypertension, and (D) DM.

Abbreviation: RCS, restricted cubic spline; BRI, body roundness index; AAC, abdominal aortic calcification; DM, diabetes mellitus.


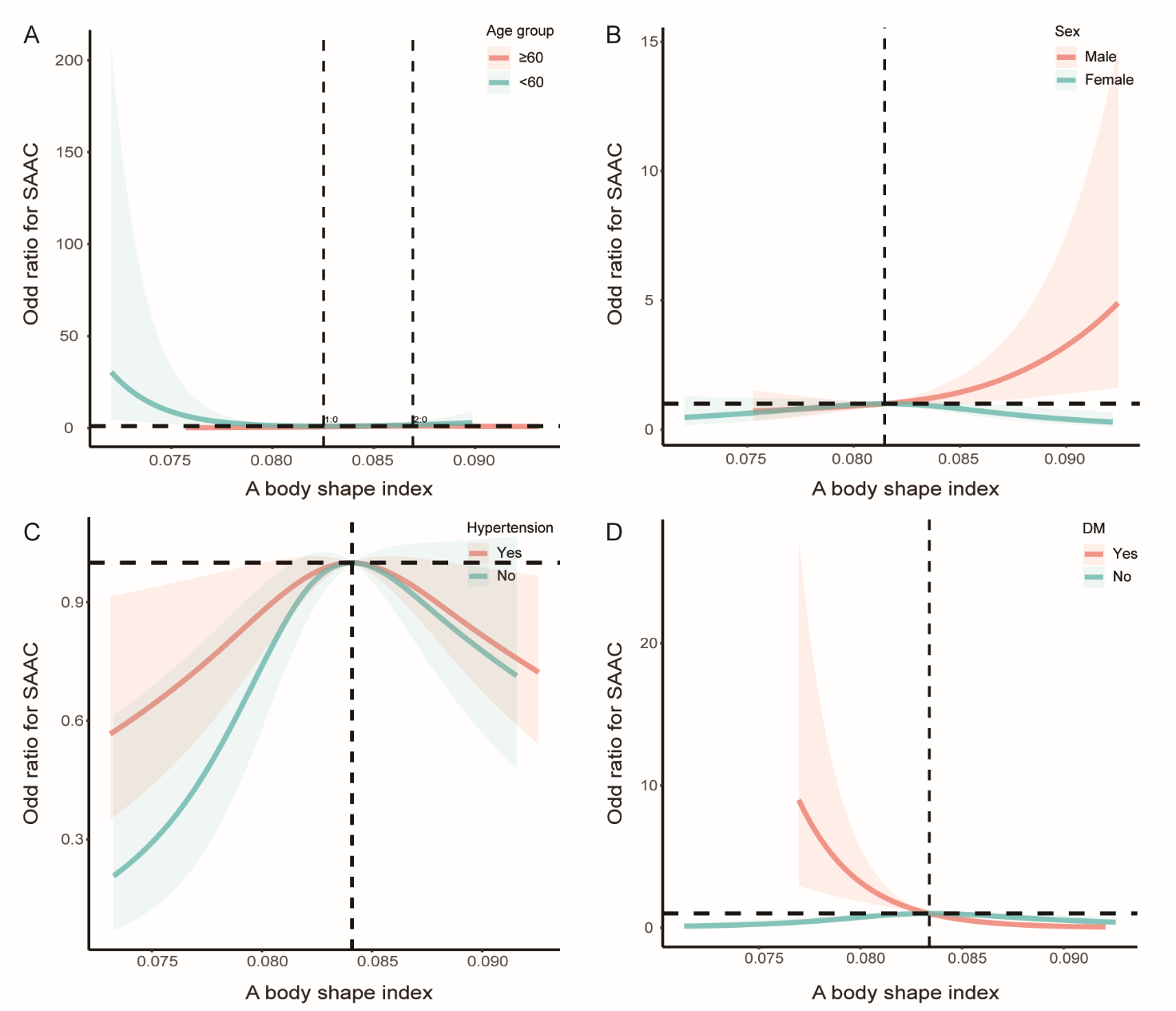


**Supplementary Fig. 3:** The RCS curve of the association between ABSI and SAAC stratified by (A) Age, (B) Sex; (C) Hypertension, and (D) DM.

Abbreviation: RCS, restricted cubic spline; ABSI, a body shape index; SAAC, severe abdominal aortic calcification; DM, diabetes mellitus.


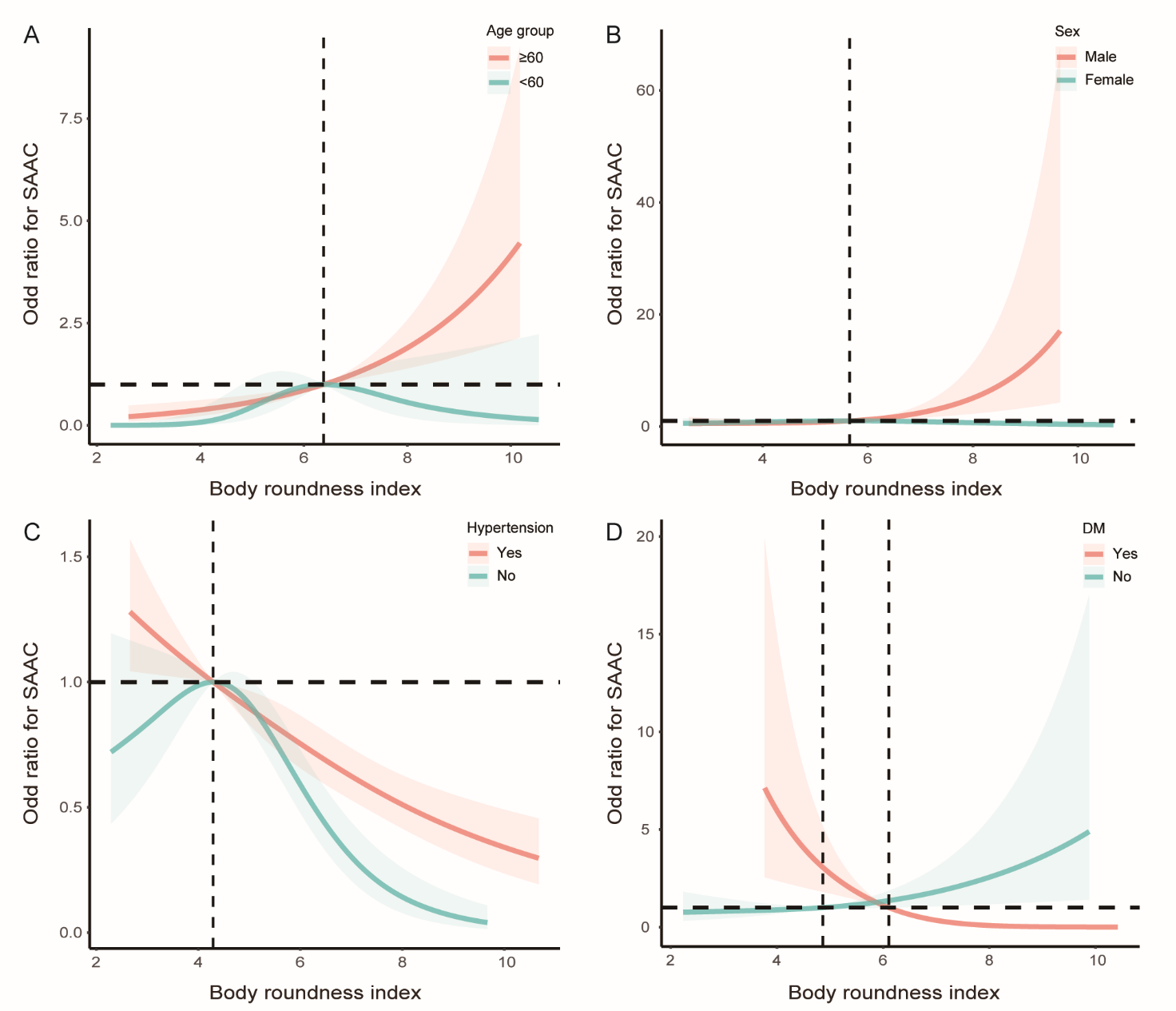


**Supplementary Fig. 4:** The RCS curve of the association between BRI and SAAC stratified by (A) Age, (B) Sex; (C) Hypertension, and (D) DM.

Abbreviation: RCS, restricted cubic spline; BRI, body roundness index; SAAC, severe abdominal aortic calcification; DM, diabetes mellitus.

Supplementary Table 1. Subgroups analysis for the associations of ABSI with the prevalence of AAC.

|  | Q1 | Q2 | Q3 | Q4 | *P* for trend | *P* for interaction |
| --- | --- | --- | --- | --- | --- | --- |
|  | OR (95%CI) | OR (95%CI) | OR (95%CI) | OR (95%CI) |  |  |
| Age |  |  |  |  |  | 0.024 |
| < 60 | 1.00 | 1.037 (0.500, 2.152) | 1.316 (0.501, 3.454) | 1.500 (0.395, 5.689) | 0.481 |  |
| ≥ 60 | 1.00 | 1.666 (0.699, 3.966) | 1.592 (0.622, 4.077) | 1.808 (0.531, 6.151) | 0.453 |  |
| Sex |  |  |  |  |  | 0.146 |
| Male | 1.00 | 1.417 (0.578, 3.476) | 1.455 (0.526, 4.025) | 2.168 (0.553, 8.507) | 0.324 |  |
| Female | 1.00 | 0.892 (0.437, 1.821) | 0.946 (0.374, 2.391) | 1.374 (0.404, 4.670) | 0.621 |  |
| Hypertension | |  |  |  |  | 0.175 |
| No | 1.00 | 0.620 (0.273, 1.406) | 0.808 (0.297, 2.200) | 0.854 (0.209, 3.490) | 0.989 |  |
| Yes | 1.00 | 1.416 (0.658, 3.045) | 1.486 (0.614, 3.594) | 2.290 (0.715, 7.337) | 0.201 |  |
| DM |  |  |  |  |  | 0.025 |
| No | 1.00 | 0.896 (0.490, 1.637) | 1.478 (0.709, 3.081) | 1.680 (0.633, 4.459) | 0.180 |  |
| Yes | 1.00 | 1.491 (0.351, 6.342) | 0.723 (0.140, 3.721) | 1.243 (0.134, 11.535) | 0.699 |  |

Abbreviations: ABSI, a body shape index; AAC, abdominal aortic calcification; DM, diabetes mellitus; Q1, 0.068–0.080; Q2, 0.081–0.083; Q3, 0.084–0.086; Q4, 0.087–0.108; OR, odd ratio; CI, confidence interval; Analysis was adjusted for age, sex, race/ethnicity, education level, marital status, family poverty-income ratio, hypertension, DM, smoker, alcohol user, coronary heart disease, congestive heart failure, angina pectoris, heart attack, and stroke, systolic blood pressure, diastolic blood pressure, mean energy intake, dietary calcium intake, dietary phosphorus intake, hemoglobin, fast glucose, fast insulin, glycohemoglobin, alkaline phosphatase, total bilirubin, serum phosphorus, and calcium, total cholesterol, triglyceride, high-density lipoprotein-cholesterol, blood urea nitrogen, uric acid, serum creatinine, estimated glomerular filtration rate.

Supplementary Table 2. Subgroups analysis for the associations of BRI with the prevalence of AAC

|  | Q1 | Q2 | Q3 | Q4 | *P* for trend | *P* for interaction |
| --- | --- | --- | --- | --- | --- | --- |
|  | OR (95%CI) | OR (95%CI) | OR (95%CI) | OR (95%CI) |  |  |
| Age |  |  |  |  |  | 0.049 |
| < 60 | 1.00 | 1.712 (0.786, 3.725) | 2.081 (0.775, 5.587) | 4.310 (0.971, 19.144) | 0.084 |  |
| ≥ 60 | 1.00 | 0.818 (0.381, 1.754) | 0.852 (0.328, 2.217) | 1.328 (0.337, 5.237) | 0.791 |  |
| Sex |  |  |  |  |  | 0.378 |
| Male | 1.00 | 1.105 (0.510, 2.391) | 1.332 (0.481, 3.689) | 2.726 (0.587, 12.655) | 0.305 |  |
| Female | 1.00 | 1.535 (0.681, 3.461) | 1.725 (0.653, 4.556) | 2.801 (0.703, 11.156) | 0.186 |  |
| Hypertension | |  |  |  |  | 0.586 |
| No | 1.00 | 0.920 (0.412, 2.057) | 0.796 (0.269, 2.350) | 0.820 (0.156, 4.321) | 0.720 |  |
| Yes | 1.00 | 1.331 (0.640, 2.768) | 1.537 (0.639, 3.696) | 2.635 (0.749, 9.274) | 0.174 |  |
| DM |  |  |  |  |  | 0.256 |
| No | 1.00 | 1.100 (0.604, 2.006) | 1.189 (0.541, 2.610) | 2.156 (0.681, 6.823) | 0.310 |  |
| Yes | 1.00 | 1.872 (0.448, 7.822) | 1.459 (0.313, 6.803) | 2.115 (0.255, 17.547) | 0.710 |  |

Abbreviations: BRI, body roundness index; AAC, abdominal aortic calcification; DM, diabetes mellitus; Q1, 1.436–4.096; Q2, 4.097–5.267; Q3, 5.268–6.642; Q4, 6.643–13.267; OR, odd ratio; CI, confidence interval; Analysis was adjusted for age, sex, race/ethnicity, education level, marital status, family poverty-income ratio, hypertension, DM, smoker, alcohol user, coronary heart disease, congestive heart failure, angina pectoris, heart attack, and stroke, systolic blood pressure, diastolic blood pressure, mean energy intake, dietary calcium intake, dietary phosphorus intake, hemoglobin, fast glucose, fast insulin, glycohemoglobin, alkaline phosphatase, total bilirubin, serum phosphorus, and calcium, total cholesterol, triglyceride, high-density lipoprotein-cholesterol, blood urea nitrogen, uric acid, serum creatinine, estimated glomerular filtration rate.

Supplementary Table 3. Subgroups analysis for the associations of ABSI with the prevalence of SAAC

|  | Q1 | Q2 | Q3 | Q4 | *P* for trend | *P* for interaction |
| --- | --- | --- | --- | --- | --- | --- |
|  | OR (95%CI) | OR (95%CI) | OR (95%CI) | OR (95%CI) |  |  |
| Age |  |  |  |  |  | 0.143 |
| < 60 | 1.00 | 0.988 (0.249, 3.923) | 1.142 (0.281, 4.644) | 1.430 (0.290, 7.045) | 0.652 |  |
| ≥ 60 | 1.00 | 3.070 (0.931, 10.122) | 3.527 (0.979, 12.714) | 2.321 (0.445, 12.096) | 0.457 |  |
| Sex |  |  |  |  |  | 0.171 |
| Male | 1.00 | 0.949 (0.117, 7.681) | 2.426 (0.339, 11.734) | 3.955 (0.302, 5.186) | 0.147 |  |
| Female | 1.00 | 2.055 (0.574, 7.358) | 1.035 (0.218, 4.907) | 0.507 (0.063, 4.107) | 0.364 |  |
| Hypertension | |  |  |  |  | 0.122 |
| No | 1.00 | 2.806 (0.475, 1.660) | 4.631 (0.759, 2.826) | 3.074 (0.508, 1.860) | 0.321 |  |
| Yes | 1.00 | 1.374 (0.405, 4.658) | 1.541 (0.407, 5.837) | 1.111 (0.194, 6.362) | 0.984 |  |
| DM |  |  |  |  |  | 0.208 |
| No | 1.00 | 2.922 (0.809, 1.055) | 4.611 (1.041, 2.042) * | 2.367 (0.324, 1.731) | 0.387 |  |
| Yes | 1.00 | 1.927 (0.106, 35.171) | 1.018 (0.044, 23.400) | 0.291 (0.005, 16.765) | 0.187 |  |

Abbreviations: ABSI, a body shape index; SAAC, severe abdominal aortic calcification; DM, diabetes mellitus; Q1, 0.068–0.080; Q2, 0.081–0.083; Q3, 0.084–0.086; Q4, 0.087–0.108; **P*<0.05; OR, odd ratio; CI, confidence interval; Analysis was adjusted for age, sex, race/ethnicity, education level, marital status, family poverty-income ratio, hypertension, DM, smoker, alcohol user, coronary heart disease, congestive heart failure, angina pectoris, heart attack, and stroke, systolic blood pressure, diastolic blood pressure, mean energy intake, dietary calcium intake, dietary phosphorus intake, hemoglobin, fast glucose, fast insulin, glycohemoglobin, alkaline phosphatase, total bilirubin, serum phosphorus, and calcium, total cholesterol, triglyceride, high-density lipoprotein-cholesterol, blood urea nitrogen, uric acid, serum creatinine, estimated glomerular filtration rate.

Supplementary Table 4. Subgroups analysis for the associations of BRI with the prevalence of SAAC.

|  | Q1 | Q2 | Q3 | Q4 | *P* for trend | *P* for interaction |
| --- | --- | --- | --- | --- | --- | --- |
|  | OR (95%CI) | OR (95%CI) | OR (95%CI) | OR (95%CI) |  |  |
| Age |  |  |  |  |  | 0.005 |
| < 60 | 1.00 | 1.896 (0.382, 9.424) | 2.352 (0.504, 1.097) | 0.158 (0.016, 1.581) | 0.187 |  |
| ≥ 60 | 1.00 | 1.846 (0.686, 4.969) | 1.617 (0.474, 5.518) | 5.161 (0.907, 29.370) | 0.177 |  |
| Sex |  |  |  |  |  | 0.230 |
| Male | 1.00 | 2.445 (0.559, 1.069) | 2.459 (0.351, 1.721) | 9.140 (0.577, 1.448) | 0.227 |  |
| Female | 1.00 | 1.572 (0.380, 6.509) | 2.394 (0.469, 1.222) | 2.776 (0.294, 2.619) | 0.327 |  |
| Hypertension | |  |  |  |  | 0.278 |
| No | 1.00 | 1.370 (0.409, 4.588) | 0.572 (0.136, 2.402) | 0.278 (0.055, 1.399) | 0.076 |  |
| Yes | 1.00 | 1.688 (0.586, 4.863) | 2.074 (0.586, 7.343) | 4.169 (0.675, 25.754) | 0.164 |  |
| DM |  |  |  |  |  | 0.387 |
| No | 1.00 | 1.057 (0.322, 3.474) | 1.252 (0.270, 5.809) | 2.118 (0.205, 2.193) | 0.578 |  |
| Yes | 1.00 | 4.796 (0.413, 5.707) | 5.533 (0.420, 7.873) | 5.829 (0.469, 8.354) | 0.198 |  |

Abbreviations: BRI, body roundness index; SAAC, severe abdominal aortic calcification; DM, diabetes mellitus; Q1, 1.436–4.096; Q2, 4.097–5.267; Q3, 5.268–6.642; Q4, 6.643–13.267; OR, odd ratio; CI, confidence interval; Analysis was adjusted for age, sex, race/ethnicity, education level, marital status, family poverty-income ratio, hypertension, DM, smoker, alcohol user, coronary heart disease, congestive heart failure, angina pectoris, heart attack, and stroke, systolic blood pressure, diastolic blood pressure, mean energy intake, dietary calcium intake, dietary phosphorus intake, hemoglobin, fast glucose, fast insulin, glycohemoglobin, alkaline phosphatase, total bilirubin, serum phosphorus, and calcium, total cholesterol, triglyceride, high-density lipoprotein-cholesterol, blood urea nitrogen, uric acid, serum creatinine, estimated glomerular filtration rate.
